# Encyclopædia Medica

**Published:** 1903-09

**Authors:** 


### Reviews of Books.

**Encyclopædia Medica.** Under the general editorship of D. CHALMERS WATSON, M.B., F.R.C.P. Edin. Vol. XII.—*Syphilis to Typhus Fever.* Pp. 528. 1902. Vol. XIII.—*Ulceration to Zinc Poisoning.* Pp. 584. Edinburgh: William Green and Sons. 1903.

These two volumes complete this great work. They both contain so much excellent material that it is impossible with the space at our disposal to do justice to them. Only a few of the contributions can be referred to.

Vol. XII. opens with an article on "Syphilis," by Mr. D'Arcy Power. He starts with a short historical review, in which he points out that originally "no great moral turpitude" was attached to the disease, as an early writer states that "in Rome this kind of illness is very partial to the priests, and especially the richest of them." The ordinary course and symptoms of syphilis are then well described, and treatment is given in detail. Attention can well be directed to the inunction method of administering mercury, which Mr. Power considers excellent under certain conditions, and "next in efficacy even if it do not surpass the plan of internal administration during the secondary stage of syphilis."

There are three separate articles on Therapeutics, viz. "Physical Therapeutics," by Mr. Ernest Flodin; "Serum Therapy," by Dr. William Murrell; and "Health Resorts," by Dr. Edmund Hobhouse. Mr. Flodin's article is by far the ongest of these, and occupies 57 pages, in which he describes very fully various exercises which can be carried out under

"genuine" medical advice. The article is illustrated by a large number of photographs showing the movements employed.

"Toxicology," by Dr. J. Dixon Mann, contains a vast amount of information in the 44 pages devoted to it. It is practically a *résumé* of his larger work, with only a few minor differences, such as classifying carbolic acid among the corrosives and a different arrangement of "poisoning by foodstuffs." The various methods of separating and estimating the poisons are treated in a very cursory manner, and all illustrative cases are omitted from this article. This latter fact naturally makes the subject less interesting; but as everything of real importance is present and well arranged, the article should prove a useful one for ready reference in cases of necessity.

The "Thyroid" is considered both from a medical and surgical aspect. The medical portion, by Dr. G. R. Murray, is well written, and a lucid description is given of the well-known morbid conditions of this gland. Mr. Frederick Page, in the surgical part, discusses the modern surgical treatment of the same diseases, and also describes malignant and other tumours of this organ.

Fifty pages are devoted to "Tuberculosis," by Dr. Theodore Shennan, senior pathologist to the Royal Infirmary, Edinburgh, and this is certainly one of the best articles in the volume. The properties and life history of the tubercle bacillus are first described very fully, and a large number of bacilli are mentioned as occasionally found in old non-tubercular cavities and in milk, etc., from which it is impossible by the ordinary staining and cultural methods to differentiate it. The relationship of bovine and human tuberculosis is discussed in a very able manner. Dr. Shennan does not agree with Professor Koch, but considers that human tuberculosis can be transmitted to cattle, especially if the latter are weakened by disease, if the culture is made more virulent by passing it through several cattle, and lastly if sputum—a mixed infection—be used for inoculation instead of a culture. He quotes many recent experiments in support of these views. The frequency of primary tubercular infection in children from the digestive tract in this country is finally discussed, and the conclusion arrived at that milk is certainly a factor in spreading the disease.

Other valuable contributions in this volume which can only be mentioned are: "Tabes Dorsalis," by Dr. F. W. Mott; "Teeth," by Mr. G. W. Watson; "Dangerous Trades," by Dr. Thomas Oliver; "Tumours," by Mr. J. Bland Sutton, and "Typhoid Fever," by Dr. Sidney Phillips.

Vol. XIII.—In this the last volume the section on Urine is particularly good. It is divided into two portions. In the first Dr. Garrod describes the "Pathological Changes in Urine" under eighteen different headings. He discusses rather minutely the clinical significance of each morbid constituent and the abnormal variability of the ordinary ingredients. The article is not made tedious by the details of complicated urinary analysis, but a large number of simple practical chemical tests are described, and fallacies of some of the commoner ones pointed out. "Bacteriological Examination," by Dr. Drysdale, comprises the second portion of this section. This is mostly compiled from recent observations, and the major portion of this short article deals with the presence of the bacillus typhosus in urine.

Mrs. Garrett Anderson's article on "Vaccination" is interesting reading, and the evidence adduced is overwhelming. She describes the vaccination systems in vogue in all the European countries, and tabulates results showing that the ratio of deaths from smallpox between the best vaccinated country, Germany, and Russia, where vaccination is not yet compulsory, is 1·1 to 463. At the end of her article Dr. Anderson points out that the Act of 1898 has had a tendency to increase successful vaccinations, but suggests many excellent amendments, among them being obligatory revaccinations, legally defined "efficient" vaccination, and that the Public Health Authorities be responsible for enforcement of the law.

An excellent contribution on "Plague," by Dr. Low, forms an appendix to this work. He considers inoculation by direct contact with infected material (not fleas) to be the usual manner of contracting this disease, and that men and rats are of about equal potentiality in spreading the same. "The Indian Plague Commission were of opinion that plague was carried through India from place to place by human agency," but . . . "the infection of the ports of this country may possibly occur by means of plague rats rather than by human agency, and a very much more difficult cause to prevent."

Nearly 100 pages are devoted to "Diseases of the Uterus," which are arranged in six sections written by different well-known gynæcologists. Other contributions which deserve more than passing notice are: "Uræmia," by Dr. Tirard; "Unconsciousness," by Dr. Hyslop; "Venereal Disease," by Mr. D'Arcy Power, dealing chiefly with soft sores and venereal warts; and "Water," by Dr. Thresh.

Dr. J. Mackenzie's article on "Visceral Pain" is quite one of the best in this volume, "the study of the pain and certain other associated symptoms arising from disease of the thoracic and abdominal organs" being thoroughly worked out. Attention also must be called to Dr. Dawson Turner's "X-rays, Finsen Light, and High-frequency Currents," in which the apparatus and manner of application of each is lucidly explained and the therapeutics discussed.

